# The relationship between shape perception accuracy and drawing ability

**DOI:** 10.1038/s41598-022-18858-6

**Published:** 2022-09-01

**Authors:** K. E. Robles, A. J. Bies, S. Lazarides, M. E. Sereno

**Affiliations:** 1 Department of Psychology, University of Oregon, Eugene, OR 97403 USA; 2 Department of Psychology, Gonzaga University, Spokane, WA 99258 USA

**Keywords:** Human behaviour, Perception

## Abstract

Accurate shape perception is critical for object perception, identification, manipulation, and recreation. Humans are capable of making judgements of both objective (physical) and projective (retinal) shape. Objective judgements benefit from a global approach by incorporating context to overcome the effects of viewing angle on an object’s shape, whereas projective judgements benefit from a local approach to filter out contextual information. Realistic drawing skill requires projective judgements of 3D targets to accurately depict 3D shape on a 2D surface, thus benefiting from a local approach. The current study used a shape perception task that comprehensively tests the effects of context on shape perception, in conjunction with a drawing task and several possible measures of local processing bias, to show that the perceptual basis of drawing skill in neurotypical adults is not due to a local processing bias. *Perceptual flexibility*, the ability to process local or global information as needed, is discussed as a potential mechanism driving both accurate shape judgements and realistic drawing.

## Introduction

The three-dimensional (3D) shape of objects and environments can be accurately determined almost instantaneously from the two-dimensional (2D) sensory information received by our retinas. This seemingly effortless capacity depends on powerful underlying neural mechanisms^1^ which are the basis for shape constancy, the ability to perceive an object as having the same physical structure and shape despite a change in viewing angle^2,3^. Shape constancy is necessary for object identification^4^ and facilitates action such as effortlessly grasping an item with proper hand position^5,6^. While estimating the objective (physical) shape of objects is useful for our daily interactions with the world, some people (individuals with realistic drawing skills) are also capable of accurately portraying a 3D scene or object as a 2D image, a process that seems inherently difficult to others. This difficulty stems from the fact that realistic drawing requires one to perceive and render the projective rather than objective shapes of objects and surfaces^7–9^.

Common errors in realistic drawing often reflect the artist’s bias towards representing the perceived physical properties of their target^10–12^. For novice artists, this “top-down” approach^13^ of utilizing additional knowledge and perspectives about the object can distort shape, size, proportion, and the presence of features. Conversely, individuals with an opposing “bottom-up” processing style are seen as possessing greater accuracy in drawing, since they render necessary line segments without the influence of additional “unseen” qualities of the object^14–16^. Individuals with Autism Spectrum Disorder (ASD)^17–19^ or those who score high on measures of autistic tendencies, as indicated by scores on the Autism^20^ or Systemizing^21^ Quotients, demonstrate a strong local processing bias associated with bottom-up processing approaches determined using Embedded Figures and Block Design tasks^17,19,20^ and have been shown to be more accurate in their realistic drawings^19^, as well as less susceptible to visual illusions that rely on varied forms of context^18,21,22^. The basis of this local processing bias has been theorized to be due to reduced integration of global contextual information (the Weak Central Coherence Theory^23,24^) or to locally-oriented enhanced perceptual processing (Enhanced Perceptual Functioning^25,26^). Other research has shown that ASD is associated with slower global processing resulting in a more automatic local processing style^27^, and that independent abilities may underlie local–global processing in neuro-typical adults^28^.

Previous research has proposed that a local processing bias in neurotypical adults also benefits realistic drawings abilities^15^. Other research, however, suggests that global processing is not impaired in participants with drawing skill^29^ and that task specific regulation of visual attention may be a primary factor that modulates the influence of context effects in drawing ability by directing attention towards the most important features of a given shape or object^28–30^ for a review, see^31^. This alternate explanation for variation in people’s drawing ability may account for findings in which drawing ability cannot be predicted by shape judgements that require contextual suppression^32,33^. Compared to non-artists, trained artists performed better on tests of projective size (but not shape) judgements^30,33,34^ and a limited-line tracing test, which indicated how well-placed participants’ focus of attention was when depicting an object. Another study^29^ showed that realistic drawing ability in adult artists and non-artists is associated with enhanced local processing as assessed by accuracy on the Group Embedded Figures task, completion times in the Block Design Task, and reduced global interference on reaction times in the Navon shape task. Since the global precedence effects in the Navon task were still extant in skilled drawers, this study also concludes that the local processing biases linked to drawing ability are not due to a reduction in global processing abilities but to an enhancement of local processing and the ability to filter out global information.

Several studies have explicitly investigated the relationship between projective shape judgements and drawing ability^2,7–9,32,33,35^. One study found that errors in perception and drawing of parallelograms were greater when context was present in line drawings^8^. Another study observed that errors in projective shape judgements of parallelograms (window frames) in the presence of context (a building) were negatively correlated with drawing accuracy, suggesting that the ability to overcome normal shape constancy mechanisms responsible for the perception of objective shape is associated with greater drawing accuracy^7^. Further studies have demonstrated a relationship between drawing skill and perceptual accuracy in estimating the projective shape of angles embedded in 3D context^9,35^. However, these results are tempered by findings in which performance on shape constancy tasks measuring estimates of projective shape do not correlate with drawing accuracy which is in itself a practical projective shape judgement task^32,33^.

Much of the early research on the estimation of shape from different viewing angles focused on contextless estimates of isolated flat surfaces. Some of these studies investigated projective estimates of shape^2^ while others investigated objective estimates^36^. Generally, it has been found that error in estimating shape increases with increasing slant, with projective judgements shifted in the direction of objective shapes (wider than correct estimates) while objective judgements are shifted in the direction of the projective shape (narrower than correct estimates) (for reviews, see^3,37,38^). Moreover, a few studies have investigated the effects of context on the perception of flat shapes, demonstrating a degree of shape constancy when making objective judgements^39,40^ and an overestimation of width (measured as an underestimation of viewing angle) when making objective judgements^7,32,33^. The only study to investigate all combinations of judgement (objective, projective) and context (present, absent) conditions demonstrated that the presence of any 3D context facilitates objective judgements but hinders projective judgements and a lack of context has the opposite effect^41^. Here we propose that it is the presence of 3D context that makes the projective judgements that are necessary for realistic drawing especially difficult.

In the present study we develop a methodology to test theories regarding the relationship between the perception of 3D shape and drawing skill to determine (1) whether drawing ability can predict shape judgement accuracy and (2) the extent to which a local perceptual bias drives differences in drawing ability. Our first question is based on the hypothesis that developing the skill of realistic drawing is accomplished by acquiring different modes for viewing objects, a process which may alter shape perception skill. This leads to the second question of whether drawing ability is influenced by perceptual tendencies naturally biased towards local processing of target details.

Skill in drawing relies on the ability to perceive and render projective shape irrespective of context. Perceptually, this can be accomplished if the perceiver ignores or suppresses contextual information such as with a local processing bias (i.e., enhanced local processing at the expense of global processing). Most research on the relationship between shape perception and drawing skill has focused on testing local processing abilities and biases, including a focus on the ability to make projective shape judgements with or without 3D context^2,7,15,32,33^. To robustly assess (1) the relationship between drawing ability and shape perception accuracy, and (2) the impact of a local processing bias on drawing ability, we use a shape perception task that comprehensively tests the effects of context on shape perception (using a completely crossed design to include all combinations of judgement (objective, projective) and context (present, absent) conditions)^41^ in conjunction with a drawing task and several potential measures of local processing bias (Autism and Systemizing Quotients^42–44^ and two new measures that compare shape perception error with and without context present). Through this comprehensive approach, we provide strong evidence regarding how perceptual performance relates to drawing ability more broadly and whether there is evidence to support a perceptual processing bias account of drawing skill.

## Results

**Figure 1:**
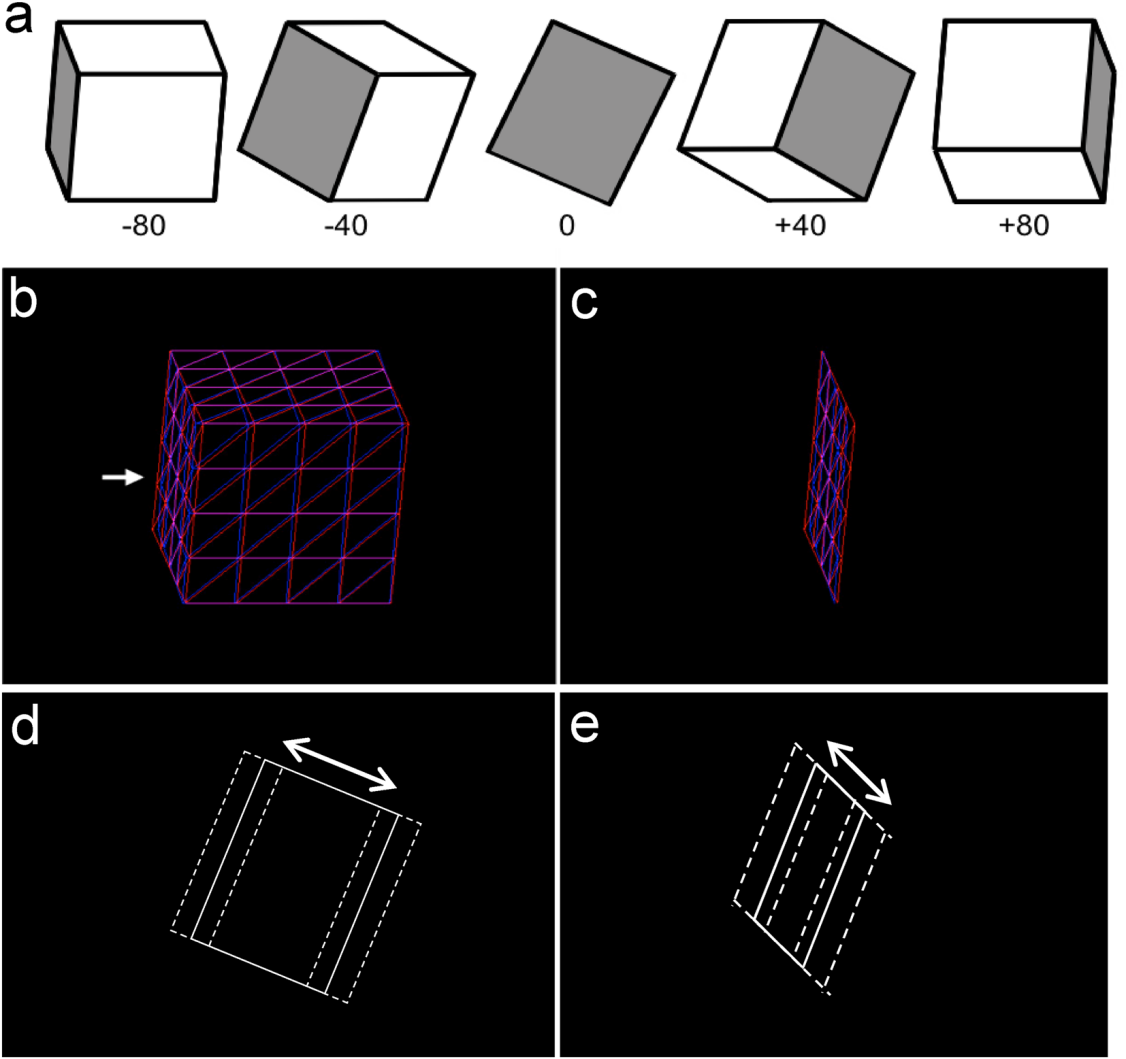
Example stimuli and response probes for the shape judgement task. (**a**) Schematic of stimuli which were polyhedrons rotated in different directions (rotated ± 40°, ± 60°, or ± 80°; the 0-degree stimulus was not presented); the face of interest is highlighted in gray. Example stimuli, which were computer-generated red/blue anaglyphs, are shown for context-present (**b**) and context-absent (**c**) blocks. Participants judged either the rectangle’s objective (physical) width, which remained constant at varying angles of rotation, or the rectangle’s projective width (the width in the picture plane). Thus, there were four block types consisting of a cross of context (present, absent) and judgement (projective, objective) types. For the context-present conditions, a white arrow (shown in **b**) indicated the face of interest that should be attended for the subsequent matching judgement. Example response probes are shown for objective (**d**) and projective (**e**) blocks. Participants had to adjust the width of the initial response parallelogram to match the previously seen stimulus. The initial response parallelogram was presented as a white outline. Example wider and narrower adjustments are indicated with dashed lines.

**Figure 2:**
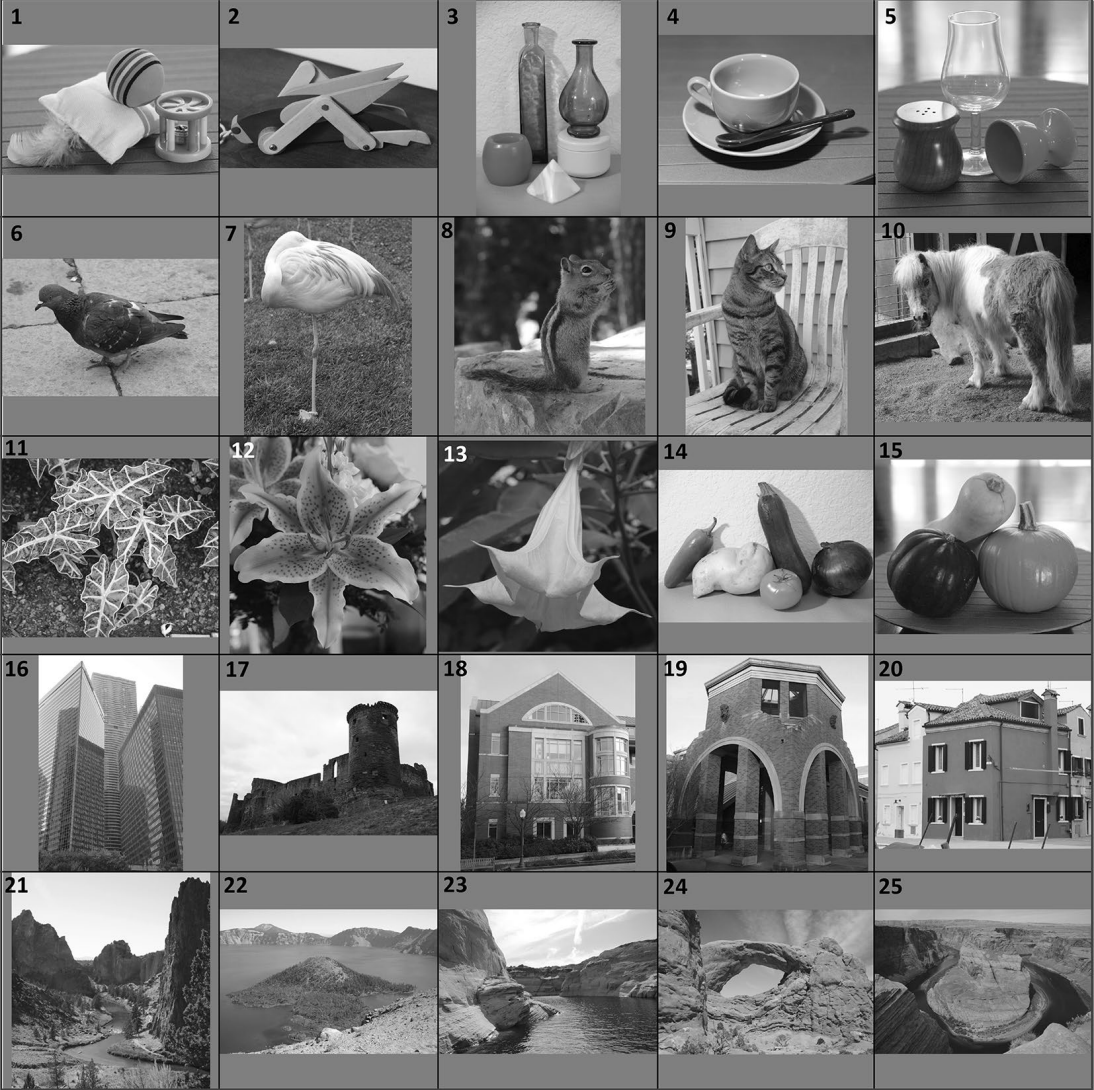
Photographs for the drawing task. Photographs were presented one at a time in random order and consisted of 5 categories—objects (still-life) (1–5), animals (6–10), plants (11–15), buildings (16–20), and natural scenes (landscapes) (21–25)—with 5 photographs from each category. All 25 images were presented for one minute each and participants were instructed to focus on accurately recreating the main lines, angles, and proportions of each image to the best of their abilities.

The experiment consisted of 3 parts (see “Methods” section) including a shape judgement task (see Fig. 1 and^41^), a drawing task (see Fig. 2), and several questionnaires: the Autism Quotient (AQ)^42^, Systemizing Quotient-Revised (SQ)^43,44^, and questions regarding previous art training and participant demographics. We first address results from the shape judgement and drawing tasks to assess how drawing ability affects shape perception, then perform a regression analysis to determine if a local processing bias can predict drawing ability. Statistical analyses were carried out in SPSS Version 28. We applied a false-discovery rate (FDR) correction to the bivariate correlation p-values shown in Table 1 to control for family-wise error rates^45,46^. These were calculated using the R software package.

**Table 1:** (a) Planned correlations among the measures of *shape perception* (projective judgements with context (PWC), objective judgements with context (OWC), projective judgements without context (PNC), and objective judgements without context (ONC)) (highlighted in italic) and between the measures of *shape perception* (PWC, OWC, PNC, ONC, and error magnitude collapsed over all shape judgement conditions (Average Error)) and *drawing accuracy* (highlighted in bold).

	Drawing accuracy	OWC	ONC	PWC	PNC	Average error
**(a)**						
Drawing accuracy	–					
OWC	***r* = − .16 (*p* = .108)**	–				
ONC	*r* = − .20 (*p* = .037*)	*r* = *.50 (p* < .001**)	–			
PWC	*r* = − .21 (*p* = .034*)	*r* = *.44 (p* < .001**)	*r* = *.43 (p* < .001**)	–		
PNC	*r* = − .24 (*p* = .013*)	*r* = *.45 (p* < .001**)	*r* = *.45 (p* < .001**)	*r* = *.63 (p* < .001**)	–	
Average error	*r* = − .26 (*p* = .007**)					–

	SQ	AQ	ΔP	ΔO		
**(b)**						
Drawing accuracy						
SQ	–					
AQ	***r* = *.10 (p* = *.339)***	–				
ΔP	*r* = *− .20 (p* = .043*)	***r* = *.15 (p* = *.129)***	–			
ΔO	***r* = *.01 (p* = *.955)***	***r* = *− .03 (p* = *.864)***	***r* = *.01 (p* = *.955)***	–		

(b) Planned correlations among the measures of *local processing bias* (difference scores between projective shape judgement conditions with and without context (ΔP), difference scores between objective shape judgement conditions with and without context (ΔO), AQ, and SQ). In 1a all four shape judgement scores (OWC, ONC, PWC, PNC) are significantly correlated with one another (italic). In addition, all shape judgements (ONC, PWC, PNC, Average Error) except the objective with context (OWC) condition are significantly correlated with Drawing Accuracy (bold). In 1b, the only significant relationship between measures of local processing bias (ΔP, ΔO, AQ, SQ) is between ΔP and SQ scores (bolditalic). *N* = 125; *p < .05; **p < .01.

### Questionnaires

To address potential biases in processing tendencies that could impact shape perception of a target object, participants completed two measures associated with ASD as an approximation of an individual’s tendency toward local processing. Participants rated their agreement with 50 statements for the AQ^42^ (with possible scores ranging from 0 to 50) and 75 statements for the SQ^43,44^ (with possible scores ranging from 0 to 150). The AQ, which assesses self-reported expression of Autism-Spectrum traits, had scores ranging from 5 to 34 (M = 17.88, SD = 4.89) with two participants with scores in the range of clinically significant autism traits. The SQ was used to quantify individual systemizing tendencies often associated with local processing tendencies and had scores ranging from 20 to 125 (M = 60.66, SD = 17.89). Out of the 125 participants, 13% of participants (n = 16) reported having taken at least one college level art course (which included options such as drawing, ceramics, and painting).

### Shape judgement task

We measured error in two ways. *Error bias* was a measure of signed magnitude (the difference between reported and correct width on each trial) where positive values indicate shape width estimates that are wider than the correct shape and negative values indicate estimates that are narrower than the correct shape; *error magnitude* was a measure of absolute magnitude (the absolute value of the difference between reported and correct width on each trial). Reported error has been converted from pixels to be degrees of visual angle. Scores for every participant were computed for each trial and averaged within each condition, thus collapsing across direction of rotation (positive/negative) and all object widths. A small size-constancy confound in the data was corrected to account for the probe stimulus being presented at 0° disparity whereas the mean depth of the face of interest was presented in front of the 0° disparity plane, placing the least rotated stimuli (± 40°) at a farther perceived distance than the most rotated stimuli (± 80°) (see^41^). Supporting size-constancy mechanisms, our data confirms that estimates of width were narrower for all angles of rotation and effects were greatest on the least rotated stimuli. Error bias, collapsed over all conditions, was on average slightly negative (− 0.12° of visual angle), and was also more negative for the smallest (− 0.16° visual angle for ± 40° rotation) compared with the medium (− 0.13° visual angle for ± 60° rotation) and largest (− 0.07° visual angle for ± 80° rotation) rotations. To correct for this small artifact, we subtracted the net bias for each angle (i.e., the mean error bias) from the individual bias scores for that angle (similar to “mean centering”, see^47^ for further discussion of the term), thus updating the error magnitude scores as well. Adjusted scores were then used to compute error magnitude, which demonstrated similar results with or without the size-constancy correction.

**Figure 3:**
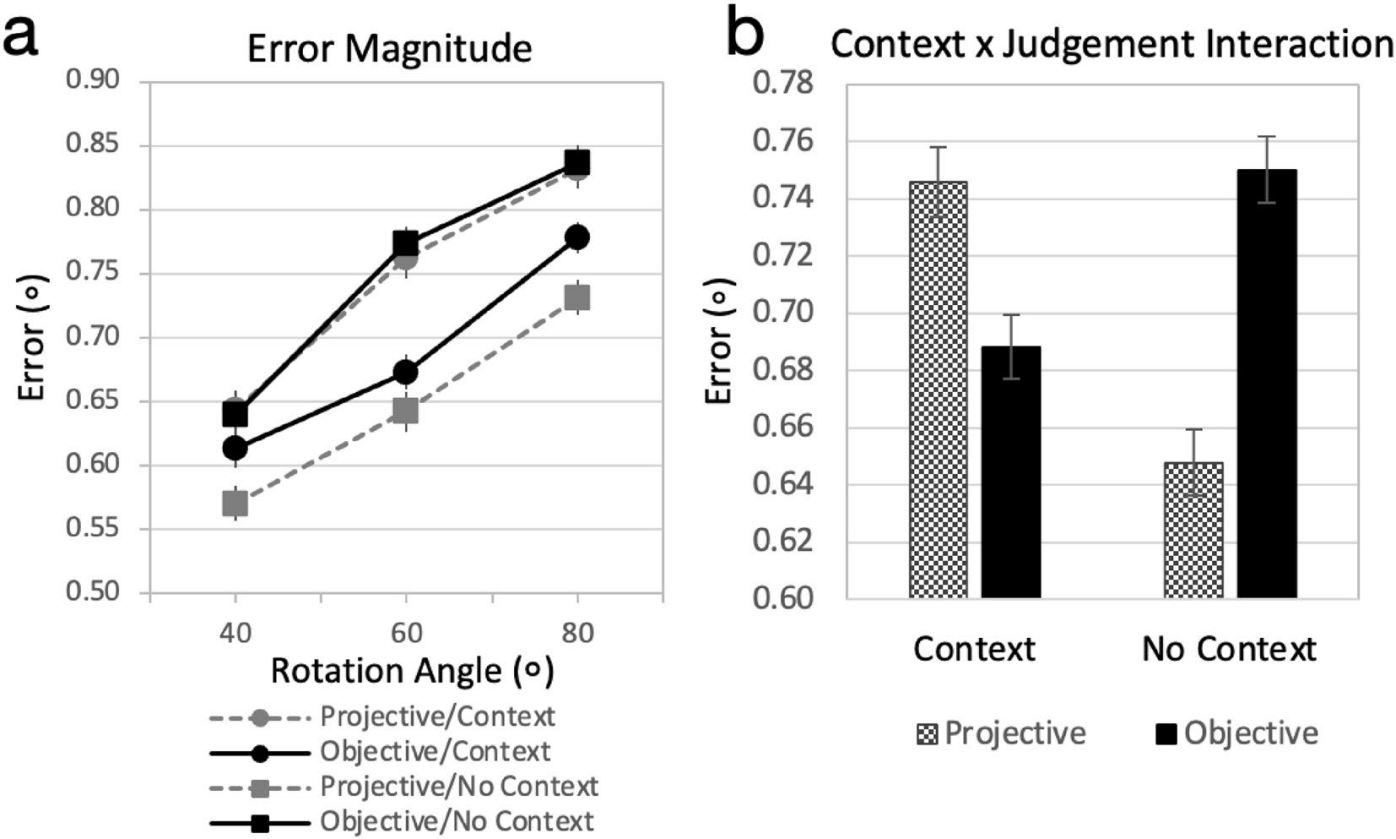
Results of the Shape Judgement Task for error magnitude data. (**a**) Error magnitude, as measured by the absolute value of the difference between reported and correct widths in terms of number of pixels and shown in degrees of visual angle, is plotted as a function of stimulus rotation angle (40°, 60°, 80°), context (context, no context), and judgement type (projective, objective). (**b**) Error magnitude is plotted (in terms of degrees of visual angle) as a function of context (context, no context) and judgement type (projective, objective). Error bars represent ± 1 *SEM*.

Data were first cleaned by removing trials in which response times were extremely short (< 500 ms) or long (> 30 s; 4.4% of trials discarded). Figure 3a plots the effects of rotation angle, context, and judgement-type on error magnitude. Performance declined as the stimulus was rotated away from a forward-facing orientation. Furthermore, performance was best for the projective/no-context and objective/context (shape constancy) conditions and worse for the objective/no context and projective/context conditions. After collapsing across angle, a 2 × 2 ANOVA with context (present, absent) and judgement (objective, projective) as within subjects factors revealed main effects of context (*F*(1, 124) = 5.34, *p* = 0.02*, *η*^2^ = 0.041) and judgement (*F*(1, 124) = 5.27, *p* = 0.02*, *η*^2^ = 0.041) and an interaction between context and judgement (*F*(1,124) = 107.66, *p* < 0.001*, η^2^ = 0.465) shown in Fig. 3b. When making objective judgements, errors were greater when context was absent (*M* = 0.75°, *CI* = [0.73°, 0.77°]) than when context was present (*M* = 0.69°, *CI* = [0.67°, 0.71°]). Whereas, when making projective judgements, errors were smaller when context was absent (*M* = 0.65°, *CI* = [0.63°, 0.67°]) than when context was present (*M* = 0.75°, *CI* = [0.72°, 0.77°])*.*

### Drawing task

**Figure 4:**
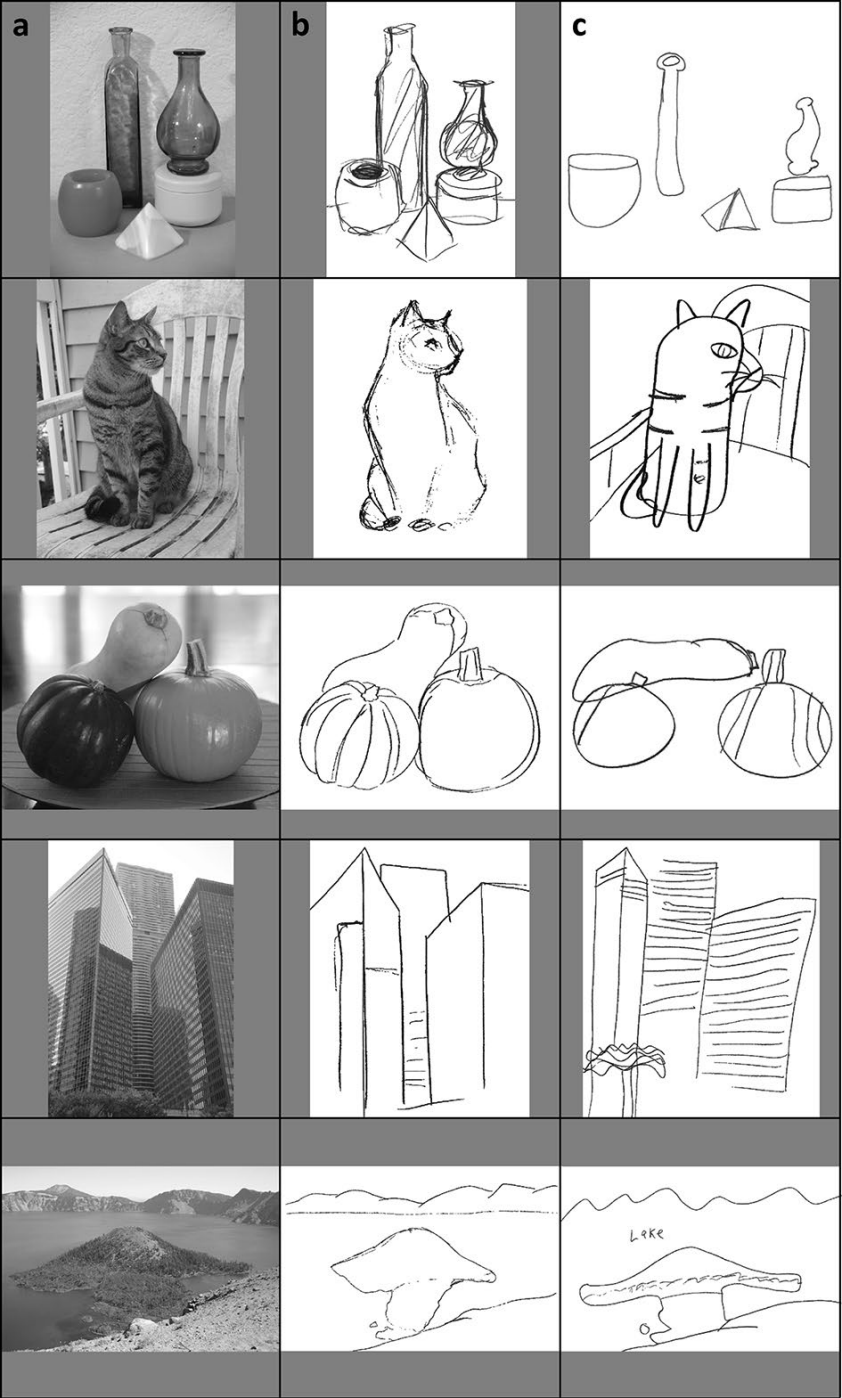
Examples of stimuli and drawings from the 5 image categories (still-life, animal, plant, buildings, and landscape). Participant renditions were rated for accuracy on a scale from 1 to 5. The images in column (**a**) show one example photo from each category. High-rated drawings (rated above the mean) for each example photo are shown in column (**b**) and low-rated drawings (rated below the mean) for each example photo are shown in column (**c**).

Participants used a pencil and sheets of blank paper to complete 1-min sketches of a series of 25 photographs, 5 photographs from each of the following categories—objects (still-lifes), animals, plants, buildings, and natural scenes (photographs shown in Fig. 2 and example drawings in Fig. 4). Participant drawings were rated independently on a scale from 1 to 5 (low to high accuracy of the renditions) by two raters. Interrater reliability was assessed using Cronbach’s alpha and was found to be adequate with an alpha of 0.79. Average overall drawing rating across participants was 2.16 (*SD* = 0.46). There was no significant difference in drawing ability (*t*(123) = -0.51, *p* = 0.61) between those who had taken a college-level drawing course (*n* = 16, M = 2.22, *SD* = 0.40), and those who had not *(n* = 109, *M* = 2.15, *SD* = 0.46). Previous drawing course experience did not predict shape judgement performance (*r* = 0.046, n = 125, *p* = 0.61), thus our measure of having taken a college-level drawing course may not be a useful indicator of drawing ability and was not included in any further analyses, though we cannot rule out that previous drawing course experiences including high school or other drawing training could have an impact on shape judgement performance.

### The effect of drawing ability on shape perception

In order to determine if the development of superior drawing skill alters shape perception accuracy, we first investigated the relationship between the different shape judgement tasks by examining two-tailed Pearson’s product-moment correlations of the error scores between the four shape perception conditions—projective judgements with context (PWC), objective judgements with context (OWC), projective judgements without context (PNC), and objective judgements without context (ONC). All four judgement conditions possess moderate correlations suggesting that greater accuracy in any one condition is related to greater accuracy in the others (Table 1a, scores highlighted in italic)*.*

**Figure 5:**
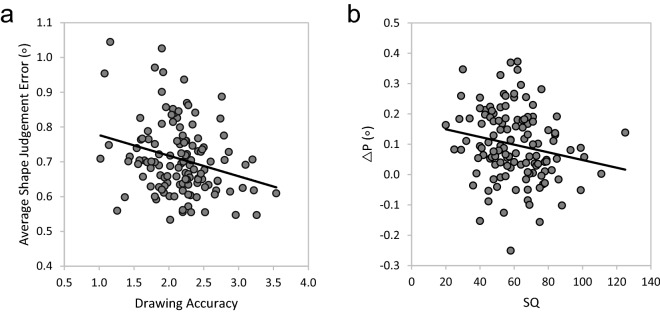
(**a**) Plot of the significant correlation between Average Shape Judgement Error and Realistic Drawing Accuracy. As realistic drawing accuracy increases, average shape judgement error decreases. (**b**) Plot of the significant correlation between two measures of local processing bias—SQ scores and difference scores between projective shape judgement conditions with and without context (ΔP in degrees of visual angle). As SQ scores increase ΔP difference scores decrease.

Next, we assessed the relationship between drawing ability and shape perception accuracy through correlations between realistic drawing accuracy and measurements of shape judgement error (Table 1a, bold). Error in three of the four shape judgement conditions (all but OWC) and average shape judgement error show moderate negative correlations with drawing ability, suggesting that greater ability to create realistic drawings is associated with overall greater accuracy in making shape judgements. A plot of the significant correlation between drawing accuracy and average shape judgement error is shown in Fig. 5a.

### Local processing bias as a predictor of drawing ability

To examine the extent to which drawing ability is influenced by an individual’s inherent local perceptual bias, which could effectively diminish the impact of surrounding context, we assessed how local processing bias is related to drawing ability. Individuals with a local processing bias should score high on the AQ as well as the SQ, and demonstrate smaller difference scores between conditions with and without context (OWC minus ONC, referred to here as “ΔO”, and PWC minus PNC, referred to here as “ΔP”) because context should have little effect on their error scores. If local processing bias is a factor which underlies drawing skill, there should be a positive relationship between the AQ and SQ scores and drawing accuracy and a negative relationship between the ΔO and ΔP scores and drawing accuracy.

We first assess the construct validity of our local processing measures through two-tailed Pearson’s product-moment correlations between the four measures of local processing bias (Table 1b, bolditalic). The only significant relationship was between the difference in projective judgement task error magnitude scores with and without context (ΔP) and SQ (Fig. 5b). Correlations among the other measures of local processing bias were not significant (Table 1b, bolditalic). As SQ scores increase in value, ΔP scores decrease with the regression line approaching “0” (no difference between projective judgement scores whether context is present or absent). For these two measures, a local processing bias is indicated by a high SQ score and low ΔP score, which explains the significant negative correlation between these two variables.

Next, we examine the relationship between drawing ability and the four potential measures of local perceptual processing bias (ΔP, ΔO, AQ, SQ) by using a regression model to predict drawing ability from the four measures of local perceptual processing bias. None of the four measures of local processing were significant predictors of variations in realistic drawing ability (Table 2). This finding suggests that the presence of a local processing bias does not account for an individual’s ability to accurately recreate a 2D representation of the 3D world through drawings.

**Table 2:** Regression analysis using the four local processing measures (AQ, SQ, ΔO, ΔP) as predictors of variation in drawing accuracy.

Df numerator	Df denominator	F	*p*	*R*^2^	95% CI for *R*^2^
4	120	.22	.926	.007	(0, .02)

Outcome variable	Predictor(s)	β	*t*	*p*	95% CI for β
Drawing accuracy	Constant	2.08	10.07	< .001	(1.67, 2.49)
	AQ	− .001	− .11	.913	(− .03, .04)
	SQ	.002	.70	.485	(− .003, .01)
	ΔO	.008	.58	.578	(− .02, .02)
	ΔP	.006	.37	.716	(− .02, .03)

None of the predictors significantly explain variation in realistic drawing ability, supporting the argument that the presence of a local processing bias is not indicative of superior realistic drawing ability.

## Discussion

Prior research on the relationship between shape perception and drawing skill has primarily focused on relating drawing skill to (1) performance on shape detection tasks (e.g., projective shape judgements) that measure the ability to overcome shape constancy mechanisms^2,7–9,32,33,35^, and (2) measures of local processing bias (AQ and SQ questionnaires; Group Embedded Figures, Block Design, and Navon tasks)^15,29^. There have been fewer tests of the relationship between global processing abilities and drawing skill^29^. The present study assesses the perceptual basis of drawing skill—specifically, the predominant *local processing bias theory* which posits that skill in drawing is due to enhanced local processing (the ability to focus on local parts of an array at the expense of global processing), in order to filter out context^15^.

To determine how individual differences in drawing ability affects shape perception, we measured perceptual processing of shape using interrelated tasks in which the utilization of context either helped or hindered performance (Fig. 1). This shape judgement experiment used a fully crossed design that varied both judgement type (projective, objective) and context (present, absent). We then related performance on the different shape judgement conditions to drawing accuracy on a drawing task (Figs. 2, 4) and to various measures of local processing bias.

The shape judgement experiment successfully replicated and extended findings from previous research^41^. Magnitude of shape judgement error increased with angle of rotation, with the least amount of error in the projective with no context and objective with context conditions (Fig. 3). These results demonstrate that the normal presence of 3D context facilitates shape constancy mechanisms (judgements of objective shape) but hinders judgements of projective shape, which are necessary for observational drawing.

Overall, we demonstrate that errors on the four shape judgement tasks were all moderately positively correlated with one another (Table 1a, italic). In addition, three of the four shape judgement conditions (all except the objective-with-context (OWC) task) along with average shape perception error were negatively correlated with drawing accuracy scores, suggesting that better realistic drawing ability is associated with more accurate shape perception in general (see Fig. 5a and Table 1a, bold). Although the relationship between the OWC judgements and drawing accuracy was not significant, it trended in the direction of a negative (higher drawing accuracy, lower OWC error) rather than a positive (higher drawing accuracy, higher OWC error) relationship as would be predicted by the local processing bias theory, since it is necessary to integrate context to achieve good performance in this condition. The fact that the negative relationship with drawing accuracy was not significant is not surprising, since OWC judgements are the basis of shape constancy and epitomize normal perceptual function. These results expand on previous work, which primarily focused on projective shape judgements in the presence of 3D context, to show that, in general, drawing skill is related to improvements in a variety of shape perception tasks and contexts.

To assess whether the presence of a local processing bias can serve as a predictor of realistic drawing ability we investigated the relationship between several possible measures of local processing bias—the AQ, SQ and two difference scores (ΔP, ΔO)—and drawing ability. Individuals with a local processing bias should score high on the AQ and SQ and have small difference scores between conditions with and without context, since context should not influence their judgements. When comparing the four measures of local processing bias, the only significant correlation was between SQ and ΔP scores (see Fig. 5b and Table 1b, bolditalic). As expected, as SQ scores increased in value ΔP scores decreased with the regression line approaching zero (no influence of context on the projective judgement scores). The lack of correlation between the AQ and SQ scores is not entirely surprising since the AQ is a much broader measure compared to the SQ which focuses entirely on systemizing tendencies (see^21^ for a discussion). As such, we expect the SQ to be a more sensitive measure of local processing. Furthermore, the lack of a significant correlation between ΔP and ΔO may be due to inherent differences in average judgement frequency—objective judgements in the presence of context typify normal perceptual processing. Therefore, one might expect projective judgements to provide a cleaner measure of the tendency to use context. Indeed, ΔP is a potentially important measure of local perceptual bias as it relates to drawing ability since projective judgements are closely tied to the perceptual processing required for drawing (judging and rendering the projective shapes of 3D surfaces). Thus, the SQ and ΔP may be more reliable measures of local processing bias and are significantly correlated in the expected direction.

Regarding the relationship between the four potential measures of local processing bias and drawing ability, a regression analysis using the four local processing measures (AQ, SQ, ΔO, ΔP) as predictors of variation in drawing accuracy indicated that none of the predictors significantly explain variation in realistic drawing ability (Table 2). This result provides evidence against a local processing bias account of drawing skill. Other research has argued that drawing ability in neurotypical adults is associated with a local processing bias as measured using the Embedded Figures and Block Design tasks^15^. However, better performance in these tasks does not indicate that the demonstrated enhanced local processing comes at the expense of global processing.

Several studies have suggested that attention may be a factor that modulates the influence of context effects in drawing ability. Examples include knowledge of which aspects of form are most important for depiction^33,34^ or the use of stored schemas for depiction of familiar objects^12^. Attention as a top-down processing mechanism has been contrasted with a bottom-up view which suggests that accurate projective judgements are a result of the reduction or suppression of perceptual transformations which result in shape constancy^7^. One such bottom-up mechanism is a local processing bias which may serve to exclude contextual information, thereby making it easier to represent projective information^15^. To account for our results, we posit a flexible processing style based on attentional mechanisms as an alternative to a bottom-up theory based on built-in perceptual biases as the perceptual basis of shape estimation in drawing skill. That is, we propose a “top-down” attention-based account of the process of overcoming shape constancy for the purpose of estimating projective shape to accurately depict 3D shape on a 2D surface. With such a processing style, drawing skill could be facilitated since context can be disregarded or utilized as needed^28,30–32^.

The present study investigated the relationship between drawing ability and perception for the purpose of answering the question of whether and how individuals with varying drawing abilities perceive the world differently from each other. Our focus was on the perception of simple 3D shapes. We utilized a methodology that included a shape perception task with a completely crossed design to fully assess the effects of different contexts and judgements on perception, in conjunction with a drawing task and measures of local processing bias. This shape judgement task closely mimicked how perceivers must use or discount contextual information to make accurate objective and projective judgements of object shape and allowed us to assess prior suggestions that individuals with superior drawing skill are generally less impacted by context. Our results provide strong evidence that the perceptual basis of drawing skill in neurotypical adults is not due to a local processing bias, suggesting instead that an attention-based flexible processing style may be driving performance on both the shape judgement and drawing tasks. These results are supported by other investigations demonstrating that superior skill in drawing is associated with enhanced local processing due to successful filtering of global information rather than a reduction in global processing abilities^29^. In general, our shape judgement task allowed us to determine that shape judgement accuracy was significantly related to drawing accuracy, suggesting that improved realistic drawing abilities may be tied to greater perceptual accuracy. Likewise, a variety of measures of local processing bias did not predict drawing accuracy. Future research using other established measures of perceptual bias (e.g., Navon tasks) can be employed to further confirm these findings. Results from the current study suggest that built-in perceptual biases do not serve as a main factor in an individual’s realistic drawing ability, and that a reliance on attentional mechanisms may instead account for variations in perceptual accuracy, a topic for future investigations.

## Methods

### Participants

To examine the extent to which artistic ability plays a role in perception of visual shape, 125 students were recruited from the University of Oregon Psychology and Linguistics Departments’ Human Subjects Pool for this study. To guarantee that our sampled population contained a more even distribution of artistic abilities, roughly half of the participants were recruited based upon their agreement with a statement in a prescreen questionnaire that read “I have an excellent ability to realistically draw things I see in the world”. Participants received either class credit for their participation or were compensated monetarily for their time. All participants indicated they had normal or corrected-to-normal vision and completed the Stereo Fly SO-001 test (Stereo Optical, Inc., Chicago, IL) to verify that they also had normal depth perception. Specifically, participants were required to score an 8 or above on the Graded Circles Test to be qualified to participate in the experiment. Informed consent was acquired following protocol approved by the Institutional Review Board at the University of Oregon, and all measures were performed in accordance with relevant guidelines and regulations for research involving human subjects as approved by this review board.

### Shape judgement task

#### Stimuli

The stimuli for the experiment consisted of 36 computer-generated red/blue anaglyphs, presented against a black background at a height of approximately 9° of visual angle. The software to generate the stimuli was written in C (utilizing OpenGL) and Tcl/Tk^48^. The stimuli were systematically rotated polyhedra drawn with an orthographic projection (see Fig. 1a for a schematic of one polyhedron). The polyhedra were first rotated downward around the x-axis by 25°, then rotated by 20° increments around the y-axis. Orientation of these figures varied from ± 80° from the frontoparallel plane, rotated around the vertical axis, including 6 different possible viewing angles (rotated ± 40°, ± 60°, or ± 80°; the 0-degree stimulus was not presented). One half (18) of the stimuli were rectangular cuboid polyhedra (Fig. 1b); the other half were isolated rectangles oriented in 3D space (Fig. 1c). Each rectangle and visible polyhedral face was completely tessellated with 32 triangles. Participants judged the width of a rectangle (the face schematically highlighted in gray in Fig. 1a). During blocks that displayed polyhedra, a small white arrow was used to indicate which of the faces should be attended for a subsequent matching judgement (Fig. 1b*).* When facing forward (0° of rotation), the depth of the polyhedra was equal to their height. The width of the polyhedra varied, with 3 different width-to-height ratios used: 0.75, 1.0 (square), and 1.25. The 36 stimuli, consisted of the combination of 6 viewing angles, 3 object widths, and 2 shapes (single rectangular face or rectangular face that is part of a polyhedron).

The response stimulus was an adjustable test shape which was a correct rendering of the face-of-interest stimulus (narrow, medium, or wide width) as a simple white outline, with its width offset by a random amount (between ± 45 pixels). For the objective judgement blocks, the test shape was the physically accurate shape, again with jitter added to the width (Fig. 1d). For the projective judgement blocks, it was an outline of the projective image (the shape in the picture plane) with added width jitter (Fig. 1e). Importantly, the added width jitter and adjustments in both cases was to the actual width of the face of interest. For the projective judgements, the resultant projective shape is what is presented to participants. This is critical methodologically because it allows for the direct comparison of objective and projective error.

#### Procedure and design

Participants sat approximately 15 inches from the computer screen and donned a pair of red-blue anaglyph glasses. Participants were instructed to complete a shape judgement task in which they were directed to make either a projective (2D width in the picture plane) or objective (the actual width of the stimulus if it were seen straight on) decision about the computer-generated red/blue anaglyphs presented. Projective decisions were described as the “seen width” of the target shape, whereas objective decisions were described as the “true width” of the target shape if it had been rotated to be viewed straight on. A tilted rectangular shape was shown with or without 3D context. Stimuli having 3D context contained the rectangular face as part of a polyhedron; stimuli without 3D context consisted of the rectangular face presented alone. Stimuli were present for 3000 ms followed by a blank screen for 1000 ms. After making the indicated judgement during the presentation of the stimuli, participants were instructed to recreate the dimensions of the target as accurately as possible by using the up and down arrow keys on the computer’s keyboard to adjust the width of a parallelogram to match the previously presented stimulus in terms of its projective or objective shape. Participants completed four types of blocks—the two different types of judgements made in two different contexts (with or without 3D context). Each block contained 54 rectangular shape target stimuli (6 angles of rotation × 3 shapes × 3 within-block repetitions). Block order, and stimulus angle and shape within each block were randomized.

#### Drawing task

Following the shape judgement task participants completed 1-min sketches using paper and a pencil of a series of 25 gray-scale photographs taken using a Nikon D3 digital camera and drawn from a larger set of photos from 5 categories: objects (still-life), animals, plants, buildings, and natural scenes (landscapes), with 5 photographs drawn from each category, see Fig. 2. Photograph presentation order was randomized and participants were instructed to draw the photographs as accurately as possible and to focus on the general contours, geometry, and correct proportions over the small details, shading and/or texture in producing their sketches. The collection of drawings from each subject was rated for level of drawing accuracy (in terms of how closely the drawing resembled the photograph primarily based on accuracy in rendering the contours and proportions) by researchers on a scale ranging from 1 to 5 (low accuracy to high accuracy).

#### Questionnaires

After the drawing task, all participants completed a multi-part questionnaire containing the Autism Quotient (AQ), Systemizing Quotient-Revised (SQ), as well as questions regarding previous art training and participant demographics. The quotients were scored based upon participants’ ratings of the degree to which the statements were representative of themselves, providing a score of autistic tendencies in general^42^ as well as systemizing tendencies in particular^43,44^. The AQ and SQ served as potential measures of possible local processing bias through their predictive relationship with ASD, commonly assumed to be associated with local processing tendencies.
